# Is competition always good? dual pacemaker management pitfalls

**DOI:** 10.1016/j.ipej.2025.07.005

**Published:** 2025-07-21

**Authors:** Ahmet Taha Sahin, Oznur Keskin, Ahmet Lutfu Sertdemir, Enes Elvin Gul

**Affiliations:** a Department of Cardiology, Beyhekim Training and Research Hospital, Konya, Turkiye; b Department of Cardiology, Necmettin Erbakan University, School of Medicine, Konya, Turkiye

**Keywords:** Pacemaker Competition, Conduction system pacing, Electrocardiographic findings, Device reprogramming

## Abstract

Alternating or variable paced QRS morphologies following pacemaker implantation is an important clinical observation. Patients undergoing pacemaker upgrade where contralateral implantation was performed, old generator usually removed. Some centers prefer either leaving the old device in the body or removal after few weeks due to risk of infection. Leaving old pacemaker in the body might lead to dangerous circumstances such as inhibiting new pacing system in a pacemaker dependent patient. This case highlights the complexities and potential complications of managing dual pacemakers.

## 1 Key messages

- This case highlights the complexities of managing dual pacemakers, where automatic reprogramming of the original device can lead to competitive pacing and distinct ECG abnormalities.
- The observation of competing pacemakers underscores the importance of careful device management and monitoring to prevent unintended interactions and complications.

## 2 Case report

A 72-year-old male with a history of hypertrophic obstructive cardiomyopathy (HOCM) and right-sided single-chamber pacemaker (VDD) implantation presented with intermittent loss of right ventricular (RV) capture. Device interrogation revealed significantly increased RV lead impedance and high threshold (1650 Ohms and 4.25 V @ 0.60 ms). Patient was 100 % pacemaker dependent with paced QRS duration of 204 ms. Patient was scheduled for new RV lead implantation.

After obtaining both verbal and written consent patient was taken to the laboratory. Right-sided venogram revealed occluded veins. Therefore, we opted to proceed with left-sided implantation and abandon the old device (Medtronic Relia, Minnesota, MN, USA). A new dual-chamber pacemaker (Enticos DR MRI, Biotronik, Germany) was implanted. Due to mild drop in the LV function (LVEF 50 %), conduction system pacing was implanted. Left ventricular septal pacing was obtained with a narrower QRS duration of 150 ms.

**Fig. 1:**
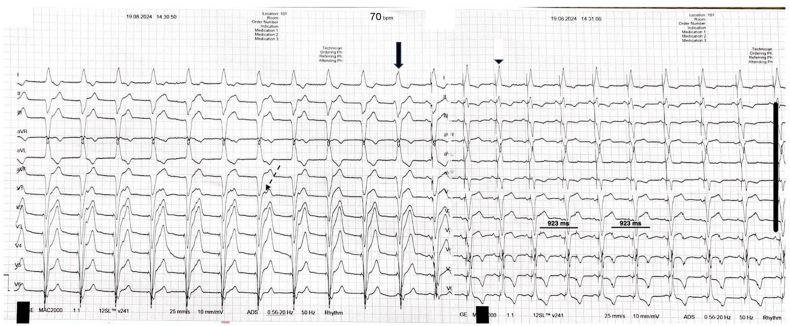
Admission 12-lead ECG revealing two parts. First part (left) showing ventricular pacing with AV dissociation (dashed arrow) and paced QRS morphology compatible with RV apical pacing (arrow) at a heart rate of 65 bpm. Second part showing sensed A and paced ventricle with QRS morphology with LV septal pacing (arrowhead) at a heart rate of 72 bpm. Note pacing spikes from the old pacemaker right on the T-waves (923 ms/65 bpm). Also note the presence of T-wave memory with conduction system pacing.

Due to risk of infection, removal of old device postponed to 4 weeks. Old device programmed as OVO to avoid device-to-device interactions. New device was programmed as DDD @ 50 bpm. One month later patient came with shortness of breath and 12-lead ECG was obtained (Fig. 1).

What is the explanation of this ECG finding?

a-) Fusion and Pseudofusion Beats
b-) Pacemaker-Mediated Tachycardia (PMT)
c-) Cross-stimulation due to lead switch
d-) Atrial lead dislodgement or inadvertent placement in the ventricle
e−) Interference from the old pacemaker

## 3 Discussion

**Fig. 2:**
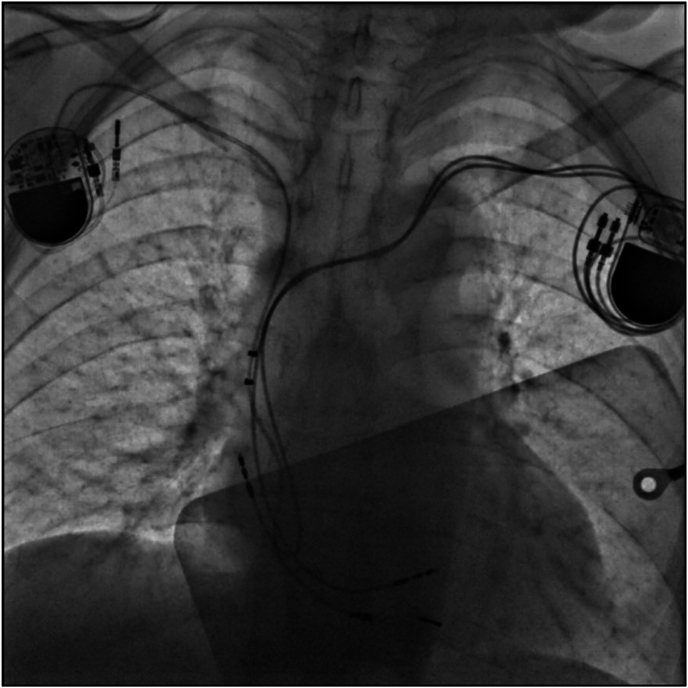
Chest X-Ray showing both devices.

In this case, the correct answer is interference between old and new pacemaker. Although old pacemaker programmed as OVO, due to triggering elective replacement indicator (ERI), device automatically switched to VVI mode @ 65 bpm. Therefore patient presented with two competing pacemakers, resulting in distinct paced QRS morphologies and spikes from the first pacemaker on the follow-up ECG. Old RV lead was placed in the apex and new one in the septum, which supports the morphoplogy of two distinct paced QRS morphologies (Fig. 2). The reactivation of the initial pacemaker in VVI mode due to its ERI status raises important questions about the underlying mechanisms.

Among the potential explanations are interference from the first pacemaker, fusion or pseudofusion beats, atrial lead dislodgement or inadvertent placement in the ventricle, and pacemaker-mediated tachycardia [[1], [2], [3]]. These factors likely contribute to the complex ECG patterns observed. However, certain mechanisms are less likely to explain the findings in this case. Although single pacemaker can create variable QRS morphologies (fused, pseudofused or pure pacing beats), in this case, due to two distinct paced QRS morphologies compatible with different lead locations (apical vs septal) rules out this explanation. The most plausible explanation is the automatic reprogramming of the first pacemaker, which led to competitive pacing. This reprogramming likely accounts for the observed phenomena, ruling out other potential mechanisms that would typically present with different ECG characteristics.

The use of a contralateral second pacemaker introduces additional complexities, particularly regarding the risk of device-related infections. However, this decision also necessitated leaving the first pacemaker in situ, which contributed to the competitive pacing issue. This underscores the need to balance infection risk with the potential for device interactions when opting for a contralateral pacemaker implantation [4].

All devices are intented to maintain support for basic pacemaker function when the battery voltage approaches ERI. In most vendors, ERI disables rate response and some features, and the mode switches to VVI mode. In Medtronic pacemakers, post ERI mode usually VVI @ 65 bpm. A key factor in this case was the automatic reprogramming of the first pacemaker from OVO to VVI mode, triggered by its ERI status. This reprogramming led to unanticipated competitive pacing, highlighting the importance of monitoring and managing device settings, especially in patients with multiple pacemakers. We speculate that the reason from switching from one QRS to another one was due to high RV threshold of the old device. This can be proven by pacing spikes (loss of capture) throughout the DDD pacing from the new device. Ensuring that the first pacemaker remains in a non-pacing mode or is appropriately reprogrammed could prevent such interactions and avoid the resultant ECG abnormalities.

In cases where a second pacemaker is necessary, ensuring that the first device is either removed or definitively deactivated can help prevent the competitive pacing observed in this patient.

## 4 Patient consent form July 01, 2025

We confirm that written informed consent has been obtained from the patient for the publication of this case report, including the use of any accompanying images and clinical data. The patient has been informed that all efforts will be made to conceal their identity, and that neither their name nor any identifiable details will be published. The patient understands that anonymity cannot be fully guaranteed, but has willingly agreed to the use of their clinical information for academic and publication purposes.

## Conflict of interest and disclosure of funding

All authors declare that the manuscript, as submitted or its essence in another version, is not under consideration for publication elsewhere, and it will not be submitted elsewhere until a final decision is made by the editors. The authors have no commercial associations or sources of support that might pose a conflict of interest. All authors have made substantive contributions to the study, and all authors endorse the data and conclusions.

## Declaration of competing interest

The authors declare that they have no known competing financial interests or personal relationships that could have appeared to influence the work reported in this paper.
